# PU-MFA: Point Cloud Up-Sampling via Multi-Scale Features Attention

**DOI:** 10.3390/s22239308

**Published:** 2022-11-29

**Authors:** Hyungjun Lee, Sejoon Lim

**Affiliations:** 1 Graduate School of Automotive Engineering, Kookmin University, Seoul 02707, Republic of Korea; 2 Department of Automobile and IT Convergence, Kookmin University, Seoul 02707, Republic of Korea

**Keywords:** 3D vision, deep-learning, point cloud, attention mechanism, point cloud up-sampling

## Abstract

Recently, research using point clouds has been increasing with the development of 3D scanner technology. According to this trend, the demand for high-quality point clouds is increasing, but there is still a problem with the high cost of obtaining high-quality point clouds. Therefore, with the recent remarkable development of deep learning, point cloud up-sampling research, which uses deep learning to generate high-quality point clouds from low-quality point clouds, is one of the fields attracting considerable attention. This paper proposes a new point cloud up-sampling method called Point cloud Up-sampling via Multi-scale Features Attention (PU-MFA). Inspired by prior studies that reported good performance at generating high-quality dense point set using the multi-scale features or attention mechanisms, PU-MFA merges the two through a U-Net structure. In addition, PU-MFA adaptively uses multi-scale features to refine the global features effectively. The PU-MFA was compared with other state-of-the-art methods in various evaluation metrics through various experiments using the PU-GAN dataset, which is a synthetic point cloud dataset, and the KITTI dataset, which is the real-scanned point cloud dataset. In various experimental results, PU-MFA showed superior performance of generating high-quality dense point set in quantitative and qualitative evaluation compared to other state-of-the-art methods, proving the effectiveness of the proposed method. The attention map of PU-MFA was also visualized to show the effect of multi-scale features.

## 1. Introduction

A point cloud is one of the most popular formats for accurately representing 3D geometric information in robotics and autonomous vehicles. Recently, the number of studies using point clouds has been increasing with the development of 3D scanners, such as LiDAR [1,2]. Along with this trend, there is an increasing demand for high-quality point clouds that are low-noise, uniform, and dense. However, the high cost of collecting high-quality point clouds remains problematic. Therefore, point cloud up-sampling, which generates a low-noise, uniform, and dense point set from noisy, non-uniform, and sparse point sets, is an interesting study.

Similar to learning-based image super-resolution studies [3,4], various learning-based point cloud up-sampling studies [5,6,7] show better performance of generating high-quality dense point set than traditional point cloud up-sampling studies [8,9]. Intuitively, the image super-resolution tasks and the point cloud up-sampling tasks are similar. Unlike the image super-resolution tasks, which process regular format images, the point cloud up-sampling tasks, which process irregular formats, require additional consideration. First, the up-sampled point set should have a uniform distribution and a dense set of points. Next, the up-sampled point set should represent the details of the target 3D mesh surface well [10].

A traditional learning-based point cloud up-sampling study usually consists of a feature extractor and an up-sampler. In addition, most studies use multi-scale features or attention mechanisms. PU-Net [11], 3PU [12], and PU-GCN [7] extract multi-scale features from sparse point sets. These studies have reported that they are excellent for generating dense point sets, but the last feature extracted by the feature extractor has a limitation in that the details of the sparse point set are diluted features because the output of each layer is used as the input of the next layer. Dis-PU [13], PU-EVA [6], and PU-Transformer [5] showed successful performance of generating high-quality dense point set using the self-attention mechanism to learn long-range dependencies between points. However, there is a limit to applying the attention mechanism with limited information because the key, query, and value of the self-attention mechanism are generated from the same input.

Focusing on these limitations, this paper proposes PU-MFA, a novel method to fuse multi-scale features and attention mechanisms. PU-MFA solves point cloud up-sampling through an attention mechanism that uses an adaptive feature for each layer. The contributions of this research are as follows:

- This paper proposes a point cloud up-sampling method of U-Net structure using Multi-scale Features (MFs) adaptively to Global Features (GFs).
- Global Context Refining Attention (GCRA), a structure for effectively combining MFs and attention mechanisms, is proposed. To the best of the authors’ knowledge, this is the first MultiHead Cross-Attention (MCA) mechanism proposed in point cloud up-sampling.
- This study demonstrates the effect of MFs by visualizing the attention map of GCRA in ablation studies.

This method was compared with various state-of-the-art methods using the Chamfer Distance (CD), Hausdorff Distance (HD), and Point-to-Surface (P2F) evaluation metrics for the PU-GAN [10] and the KITTI [14] dataset. As a result, the effectiveness of this method was confirmed by showing better performace at generating dense point set.

## 2. Related Work

### 2.1. Optimization-Based Point Cloud Up-Sampling

Various optimization-based studies have been performed to generate a dense set of points from a sparse set. Alexa et al. solved up-sampling by inserting new points into the Voronoi diagram of the local tangential space computed based on the moving-least-squares error [8]. Lipman et al. explained the up-sampling using the Locally Optimal Projection (LOP) operator [9]. In this study, the points were re-sampled by using $L_{1}$ norm. Huang et al. up-sampled a noisy and non-uniform set of points using an improved LOP that is a weighted LOP [15]. Later, Huang et al. proposed an advanced method called Edge-Aware Re-sampling of a set of points (EAR). The EAR first re-samples the edges and then uses edge-aware up-sampling to resolve the up-sampling [16].

### 2.2. Learning-Based Point Cloud Up-Sampling

With the successful performance of learning-based image super-resolution, many studies have proposed a learning-based point cloud up-sampling method.

As with image analysis, many studies have used MFs in point cloud up-sampling. PU-Net [11], the first attempt at deep learning for point cloud up-sampling, showed good performance at generating high-quality point set by extracting MFs through hierarchical feature learning and interpolation based on the framework of PointNet++ [17]. 3PU [12] performed well using MFs via an Intra-Level Dense connection and Inter-Level Skip connection. PU-GCN [7] uses MFs extracted by Inception DenseGCN. In this study, Inception DenseGCN could effectively extract MFs with an InceptionNet-inspired structure [18].

Because of the advantages of learning the long-range dependency of the self-attention mechanism, it is used in various point cloud up-sampling studies. In PU-GAN [10], generators are trained using discriminators that apply a self-attention mechanism. Pugeo-Net [19] showed good performance at generating high-quality dense point set by using it in Feature Recalibration. PU-EVA [6] showed successfully generating up-sampled point set using an EVA Expansion Unit with the mechanism. Dis-PU [13] performed well using the Local Refinement Unit with self-attention applied to the generated point set. PU-Transformer [5], which applied the transformer structure for the first time in point cloud up-sampling, uses Shifted Channel MultiHead Self-Attention to show the state-of-the-art performance of generating high-quality point set.

## 3. Problem Description

**Figure 1:**
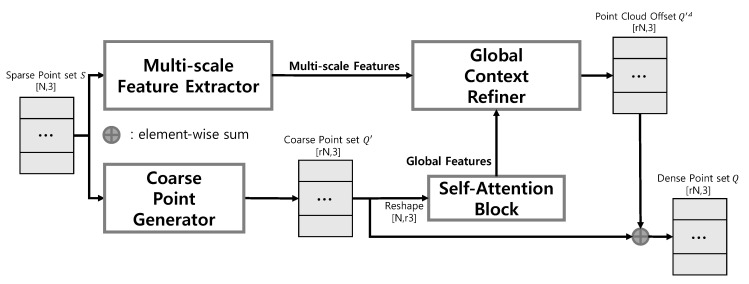
Illustration of an overview of the proposed method.

Given an unordered sparse point set $S={\left\{s_{i}\right\}}_{i=1}^{N}$ of *N* samples, we aim to generate low-noise, uniform, and dense point set $Q={\left\{q_{i}\right\}}_{i=1}^{rN}$ using $D={\left\{d_{i}\right\}}_{i=1}^{rN}$ as Ground Truth (GT), where *N* is the input patch size and *r* is the up-sampling ratio. Figure 1 shows the problem description of this study. Also, Table 1 summarizes the definitions of symbols to be used.

## 4. Method

**Figure 2:**
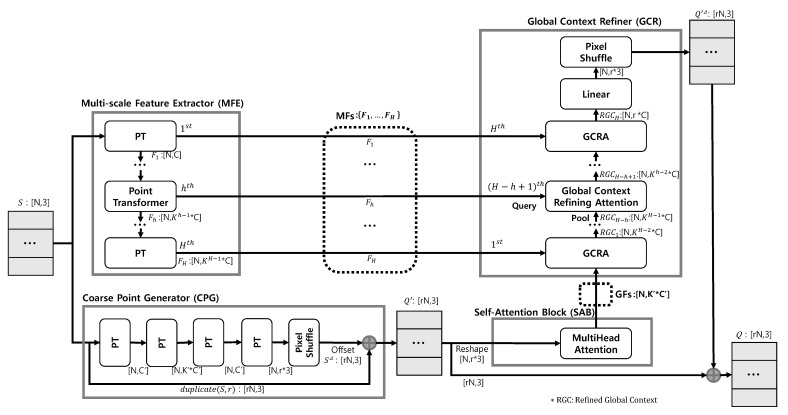
Illustration of the proposed framework. Here, 3 is the coordinate dimension, and *H* is the depth of the layer. In Multi-scale Feature Extractor (MFE) and Global Context Refiner (GCR), *C* is the channel, and *K* is the expansion ratio. In Coarse Point Generator (CPG) and Self-Attention Block (SAB), C′ is the channel and $K^{\prime }$ is the expansion ratio.

This method consists of a Multi-scale Feature Extractor (MFE), Global Context Refiner (GCR), Coarse Point Generator (CPG), and Self-Attention Block (SAB). As shown in Figure 2, MFE extracts MFs, an adaptive feature for use in GCR. GCR uses MFs to refine GFs adaptively and finally produce $Q^{\prime }{}^{\Delta }$, where $Q^{\prime }{}^{\Delta }$ is defined as $Q^{\prime }{}^{\Delta }={\left\{q_{i}^{\prime }{}^{\Delta }\right\}}_{i=1}^{rN}$. CPG generates $Q^{\prime }$ from *S* and SAB extracts GFs from $Q^{\prime }$, where $Q^{\prime }$ is defined as $Q^{\prime }={\left\{q_{i}^{\prime }\right\}}_{i=1}^{rN}$. Based on the definitions of $Q^{\prime }$ and $Q^{\prime }{}^{\Delta }$, *Q* is formulated as Equation (1), where ⊕ is an element-wise sum.

(1) $$\begin{array}{c} Q=Q^{\prime }⊕Q^{\prime }{}^{\Delta }\left(⊕:\mathrm{element}-\mathrm{wise}\mathrm{sum}\right) \end{array}$$

### 4.1. Multi-Scale Feature Extractor

Because the GFs extracted from $Q^{\prime }$ via SAB is a feature extracted from a set of points in which geometric information about the original input *S* is diluted, MFE using Point Transformer (PT) [20], an advanced point cloud analysis technique, extracts MFs from *S*. As shown in Figure 2, the MFE consists of *H* PT, and the set of point-wise features extracted from the $h^{th}$ PT is $F_{h}\in R^{N\times K^{h-1}C}$. MFs are the set of $F_{h}$ extracted from all layers of the MFE. The extracted MFs and $F_{h}$ are formulated as in Equation (2), where $f_{i}^{h}$ is a point-wise feature extracted from the $h^{th}$ PT.

(2) $$\begin{array}{cc} \; & F_{h}={\left\{f_{i}^{h}\right\}}_{i=1}^{N},MFs={\left\{F_{h}\right\}}_{h=1}^{H} \end{array}$$

#### Point Transformer

PT consists of two elements. The first is the K-Nearest Neighbor (KNN), and the second is the Vector Self-Attention (VSA) mechanism. At the $h^{th}$ PT, the point-wise feature $f_{i}^{h-1}$ of point $s_{i}\in S$ is updated to $f_{i}^{h}$ through VSA, which uses $s_{i}$ and $patch_{i}$ as the inputs. The $patch_{i}$ is generated through KNN using $s_{i}$ as the input. This operation works on all points in *S*, updating the point-wise feature of all points [20]. This is formulated in Equation (3), where patch_size is the size of KNN’s neighbor size.

(3) $$\begin{array}{cc} \; & patch_{i}=KNN\left(s_{i},patch\_size\right)\\ \; & f_{i}^{h}=\sum_{p_{k}\in patch_{i}}VectorSelfAttention\left(s_{i},p_{k}\right)\\ \; & \left(s_{i}\in S,i\in \left\{1,2,...,N\right\}\right) \end{array}$$

**Figure 3:**
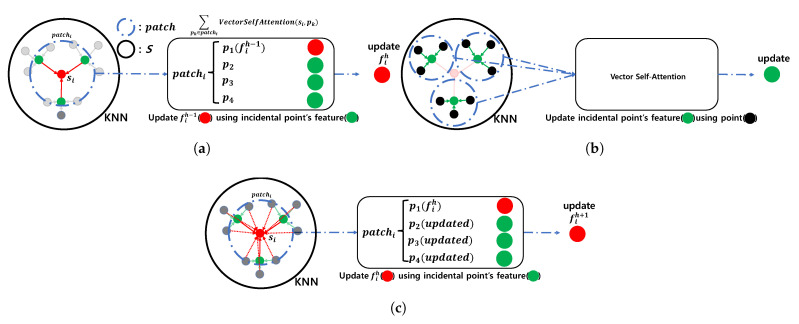
Illustration of KNN and VSA in PT. (**a**) $h^{th}$ Point Transformer layer with $s_{i}$. (**b**) $h^{th}$ Point Transformer layer with incidental points. (**c**) ${\left(h+1\right)}^{th}$ Point Transformer layer with $s_{i}$.

Inspired by this operation, this considered $patch_{i}$ is equivalent to the CNN’s kernel. In CNN, even if the kernel of the CNN is fixed, a deeper layer, means a wider receptive field. Therefore, even if the patch size of the KNN in PT is fixed, the deeper the layer, the more $s_{i}$ can interact with a wider range of points. Figure 3 is an example with a KNN patch size of four. In Figure 3a, when the $h^{th}$ PT updates $f_{i}^{h-1}$ to $f_{i}^{h}$, VSA is performed on $patch_{i}$, which is composed of $s_{i}$ and incidental points, to update $f_{i}^{h-1}$. In Figure 3b, the $h^{th}$ PT updates each feature by performing a VSA for each patch in all incidental points. In Figure 3c, the ${\left(h+1\right)}^{th}$ PT updates $f_{i}^{h}$ to $f_{i}^{h+1}$ by performing VSA using $patch_{i}$ similar to the $h^{th}$ PT. However, the ${\left(h+1\right)}^{th}$ PT updates $f_{i}^{h}$ to $f_{i}^{h+1}$ using a wider receptive field than the receptive field of the $h^{th}$ PT because the features of the incidental points of the ${\left(h+1\right)}^{th}$ PT are updated by the $h^{th}$ PT. This operation allows the MFE to extract the MFs effectively.

### 4.2. Global Context Refiner

Because GCR and MFE are U-Net [21] structures, they are composed of *H* GCRA. GCRA effectively refines GFs by querying MFs, which is adaptive geometric information applied to each layer. As shown in Figure 2, the ${\left(H-h+1\right)}^{th}$ GCRA generates $RGC_{H-h+1}\in R^{N\times K^{h-2}C}$ by using $F_{h}\in R^{N\times K^{h-1}C}$ as a query and $RGC_{H-h}$ as a pool. However, $R^{N\times r3}$ was used instead to prevent $RGC_{H}$ from becoming $R^{N\times \frac{C}{K}}$. After refining the GFs, the linear layer was used to perform the transformation. PixelShuffle was then used to generate the $Q^{\prime }{}^{\Delta }\in R^{rN\times 3}$.

#### Global Context Refining Attention

**Figure 4:**
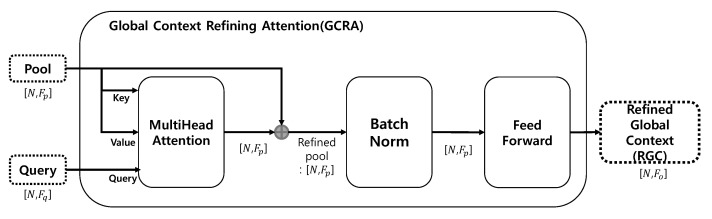
Illustration of Global Context Refining Attention (GCRA). $F_{p}$ is the pool input channel, $F_{q}$ is the query input channel, and $F_{o}$ is the output channel.

Inspired by Skip-Attention [22], which acts as a communicator between the encoder and decoder, GCRA uses MFs and GFs to apply the MCA mechanism. In various studies, self-attention mechanisms are used to extract the features of point sets or to generate up-sampling point sets [5,10]. However, the self-attention mechanism is limited because it uses only limited information due to the structure in which key, query, and value are generated from the same input. With these limitations in mind, GCRA in the *H* hierarchy uses GFs $\in R^{N\times \;K^{\prime }C^{\prime }}$ as the pool (key, values) and MFs as the queries, progressively refining the GFs through MCA. GCRA consists of MCA [23], Batch Normalization (BN) [24], and Feed Forward. As shown in Figure 4, the output shape of applying MCA using the query and pool is $R^{N\times F_{p}}$. The pool was then refined by adding the pool and the MCA output. The BN was used for stable training after addition. Feed Forward transforms the output of the BN and produces a Refined Global Context (RGC) $\in R^{N\times F_{o}}$.

### 4.3. Coarse Point Generator

CPG generates $Q^{\prime }$. In CPG, PT [20] and PixelShuffle [5,25] generate $S^{\prime }{}^{\Delta }$ from *S*, where, $S^{\prime }{}^{\Delta }$ is defined as $S^{\prime }{}^{\Delta }={\left\{s_{i}^{\prime }{}^{\Delta }\right\}}_{i=1}^{rN}$. The structure of CPG consists of four layers, such as the structure of the 3PU’s Feature Extraction Unit [12]. As shown in Figure 2, to make the final output into 3D coordinates, first, PT was first used to expand the features, and then gradually reduce them. Subsequently, PixelShuffle generates 3D coordinates using those features. $Q^{\prime }$ is generated through the element-wise sum of the generated $S^{\prime }{}^{\Delta }$ and $duplicate\left(S,r\right)\in R^{rN\times 3}$. This process is formulated as Equation (4).

(4) $$\begin{array}{cc} \; & duplicate\left(S,r\right)={\left\{\overset{r\mathrm{times}}{\overset{︷}{s_{i},\ldots ,s_{i}}}\right\}}_{i=1}^{N}\\ \; & Q^{\prime }=duplicate\left(S,r\right)⊕S^{\prime }{}^{\Delta } \end{array}$$

### 4.4. Self-Attention Block

Inspired by self-attention, which learns long-range dependency [23], we use MultiHead Self-Attention (MSA) was used to extract the GFs from $Q^{\prime }$. As shown in Figure 2, the shape of $Q^{\prime }$ was changed from $R^{rN\times 3}$ to $R^{N\times 3r}$, and the coordinates of $Q^{\prime }$ were used as features of the original point set *S*. The GFs $\in R^{N\times K^{\prime }C^{\prime }}$ was then extracted using the changed shape $Q^{\prime }$ as the input to the MSA.

## 5. Experimental Settings

### 5.1. Datasets

All methods were trained using the most popular PU-GAN [10] dataset in these experiments and evaluated using the PU-GAN dataset and the KITTI [14] dataset. The PU-GAN dataset was a synthetic point cloud dataset produced from 147 3D meshes, and the KITTI dataset was a real-scanned point cloud dataset collected using real LiDAR.

The training phase used 120 3D meshes from the PU-GAN dataset. All patches were generated via the Poisson disk sampling after converting the original mesh to a point cloud, just like the patch-based up-sampling approach. The sampling resulted in 24,000 input-output pairs.

In the evaluation phase, 27 3D meshes from the PU-GAN dataset were converted into point clouds to test the synthetic point up-sampling, and the real-scanned point up-sampling test was performed using the KITTI dataset. The generated patches should cover all point sets when evaluating the synthetic point cloud and real-scanned point cloud up-sampling. After merging each up-sampled patch, the up-sampled point set was reconstructed by farthest point sampling. More details can be found at study in PU-GAN [10]. This dataset was downloaded and used from https://github.com/liruihui/PU-GAN (accessed date: 12 July 2022).

### 5.2. Loss Function

In most point cloud reconstruction methods, CD is used as the loss function [22,26,27]. However, it was confirmed empirically that the Using Density-Aware Chamfer Distance as loss function showed good performance at point cloud reconstruction, considering the uniformity of the points set on the CD [28]. Therefore, the total loss was formulated as Equation (5), where α is linearly interpolated from 0.1 to 1 during training and ${∥\cdot ∥}_{2}$ is $L_{2}$ norm.

(5) $$\begin{array}{cc} \; & \mathrm{Loss}\left(Q^{\prime },Q,D\right)=L_{\mathrm{CD}}\left(Q^{\prime },D\right)+\alpha \times L_{\mathrm{DCD}}\left(Q,D\right)\\ \; & L_{\mathrm{CD}}\left(Q^{\prime },D\right)=\frac{1}{\left|Q^{\prime }\right|}\sum_{x\in Q^{\prime }}\underset{y\in D}{min}{∥x-y∥}_{2}+\frac{1}{\left|D\right|}\sum_{y\in D}\underset{x\in Q^{\prime }}{min}{∥y-x∥}_{2}\\ \; & L_{\mathrm{DCD}}\left(Q,D\right)=\frac{1}{\left|Q\right|}\sum_{x\in Q}^{}\underset{y\in D}{min}\left(1-e^{-{∥x-y∥}_{2}}\right)+\frac{1}{\left|D\right|}\sum_{y\in D}^{}\underset{x\in Q}{min}\left(1-e^{-{∥y-x∥}_{2}}\right) \end{array}$$

### 5.3. Metric

This study evaluated the method using CD, HD, and P2F metrics, as in prior studies [5,6,13]. CD is a metric that measures the similarity between a set of GT points and a set of predicted points for each point, and HD is an evaluation metric that measures the outliers in a set of predicted points based on a set of GT points. P2F is an index that measures the similarity between the original mesh and the predicted point set and measures the quality of the predicted point set. The parameter complexity was also measured by measuring the number of parameters. For all metrics, a lower the number, meant better performance.

### 5.4. Comparison Methods

The proposed method was compared with three state-of-the-art methods: Dis-pu [13], PU-EVA [6], and PU-Transformer [5] to validate the method. For an exact comparison, all methods were implemented using pytorch [29] version 1.7.0 on Ubuntu 20.04 and trained on the same Intel i9-10980XE CPU and NVIDIA TITAN RTX environment.

### 5.5. Implementation Details

All methods for the experiment were trained with a batch size of 64 for 100 epochs, and the Adam [30] optimizer with a learning rate of 0.0001 was used. The patch size of KNN used in PT is set to 20 as in PU-Transformer [5]. Rotation, scaling, random perturbation, and regularization were applied to the training dataset. as in prior studies [10,11]. The up-sampling ratio *r* was four and the input patch size *N* was 256. The CPG’s $C^{\prime }$ and $K^{\prime }$ were 32 and 8, respectively. For MFE and GCR, *C* and *K* were 16 and 4, respectively. The layer depth of MFE and GCR, *H*, was four. The head number of MCA and MSA was set to eight, as in the prior study [23]. Here, the head is used to learn different perspectives in Multihead Attention.

## 6. Experimental Results

Dis-PU [13], PU-EVA [6], and PU-Transformer [5], and the present method were compared using the PU-GAN [10] and the KITTI [14] datasets.

### 6.1. Results on 3D Synthetic Datasets

Table 2 lists the quantitative performance comparisons for ×4 and ×16 up-sampling. ×4 up-sampling sampled 2048 points to 8192 points. ×16 up-sampling sampled 512 points to 8192 points by repeating the ×4 up-sampling twice. As shown in Table 2, the present method showed good performance of generating high-quality point set compared to the other state-of-the-art methods. Presented method has the best value in the evaluation metric compared to other methods with similar parameter complexity. As shown in Table 3, the time complexity of the proposed method is similar to that of other methods.

**Figure 5:**
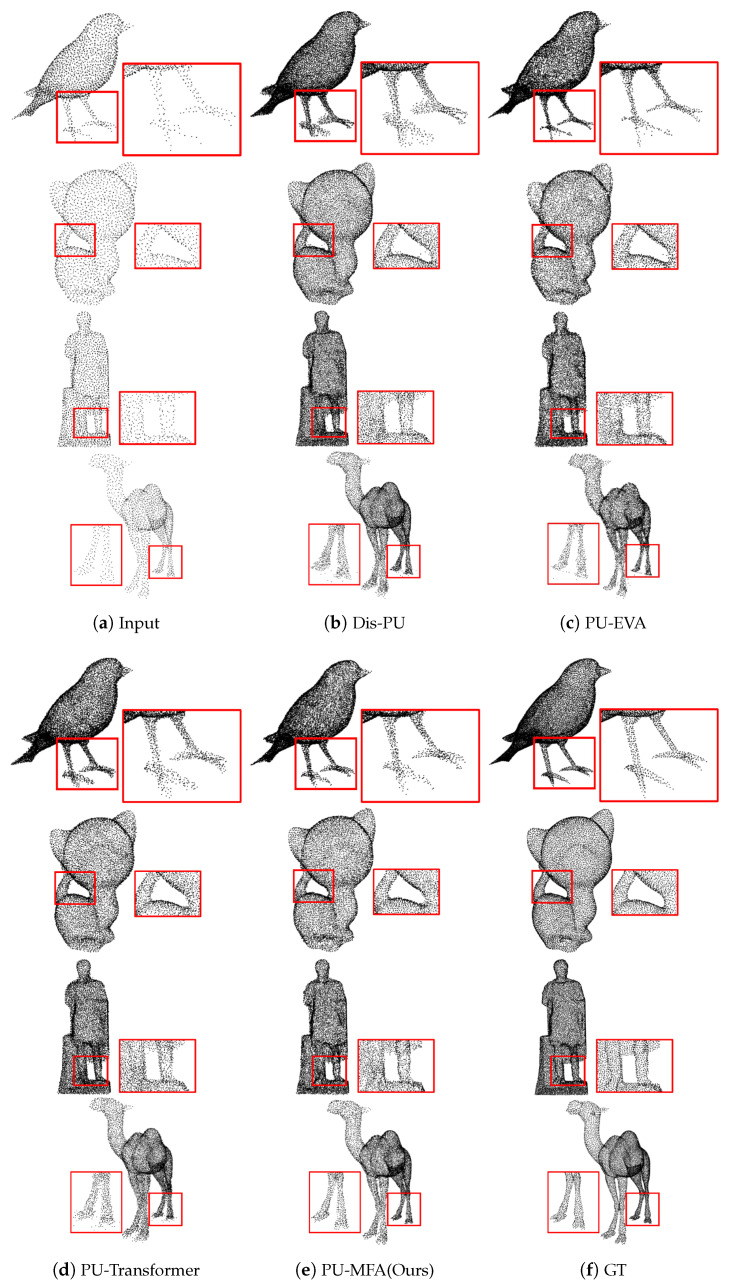
Visualization result of ×4 up-sampling on PU-GAN dataset (tubular objects).

**Figure 6:**
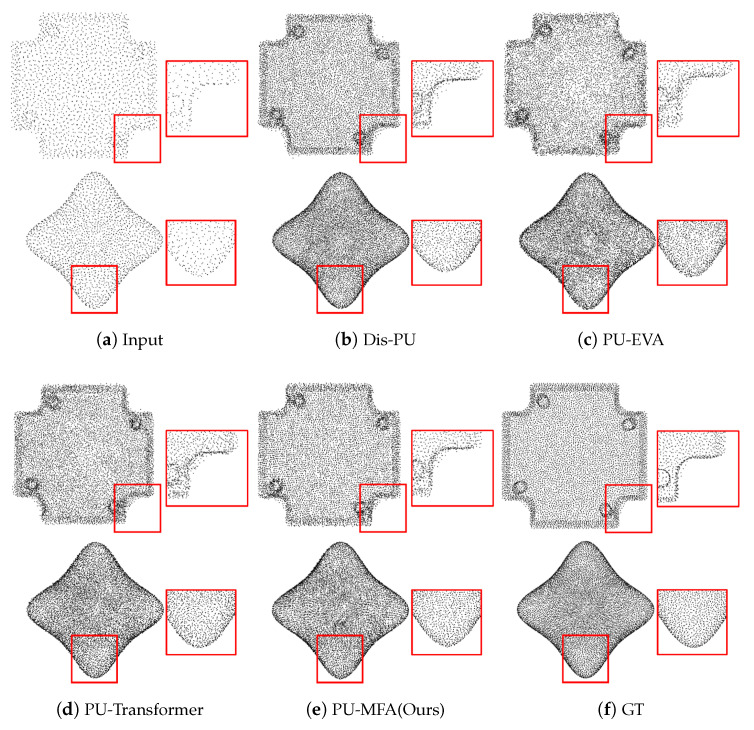
Visualization result of ×4 up-sampling on PU-GAN dataset (non-tubular objects).

**Figure 7:**
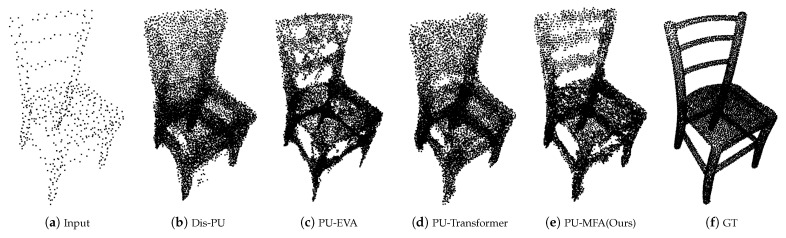
Visualization result of ×16 up-sampling of PU-GAN dataset.

Figure 5 and Figure 6 present the visualization result of ×4 up-sampling, and Figure 7 is the visualization result of ×16 up-sampling. Figure 5b–d, show a set of points representing a tubular object, such as a bird’s leg, the space between the kitten’s body and tail, a statue’s leg, and a camel’s hoof with unclear boundaries. However, Figure 5e shows low-noise and clear boundaries. Also, Figure 6b–d, show the set of points representing non-tubular objects with noise the LP rear control cover and star. However, Figure 6e shows low noise in non-tubular objects.

In Figure 7b,d, the chair back does not represent the original shape well, and Figure 7c maintains the shape to some extent, but there is considerable noise. On the other hand, Figure 7e has relatively little noise and represents the original shape well.

### 6.2. Results on Real-Scanned Datasets

**Figure 8:**
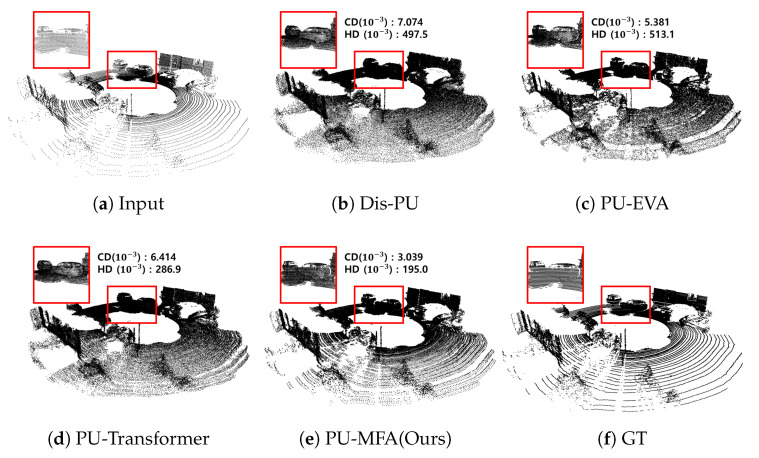
Visualization result of ×4 up-sampling of the KITTI dataset.

Dis-PU, PU-EVA, PU-Transformer, and the present method were evaluated using the KITTI dataset for ×4 up-sampling. Figure 8 shows ×4 up-sampling. In Figure 8b–d, the boundary between the window and the door of the vehicle was unclear. However, Figure 8e generated by the present method, showed that the boundary was clearer.

### 6.3. Ablation Study

This method, was evaluated by performing various ablation studies using the PU-GAN dataset.

#### 6.3.1. Effect of Components

To demonstrate the effectiveness of the contribution, four cases were divided into ablation studies. The cases were as follows: Case 1 was a structure using GCR, CPG, and SAB, with the MultiHead Attention (MHA) of GCR and SAB consisting of self-attention with one head. Case 2 was a structure changed from Case 1 to eight heads. Case 3 was a structure using GCRA composed of MCA by adding MFE to Case 2, where the query of all GCRA becomes $F_{4}$, the final output of MFE. Case 4 was PU-MFA. As shown in Table 4, all contributions affected the method performance of generating point set.

#### 6.3.2. Multi-Scale Features Attention Analysis

**Figure 9:**
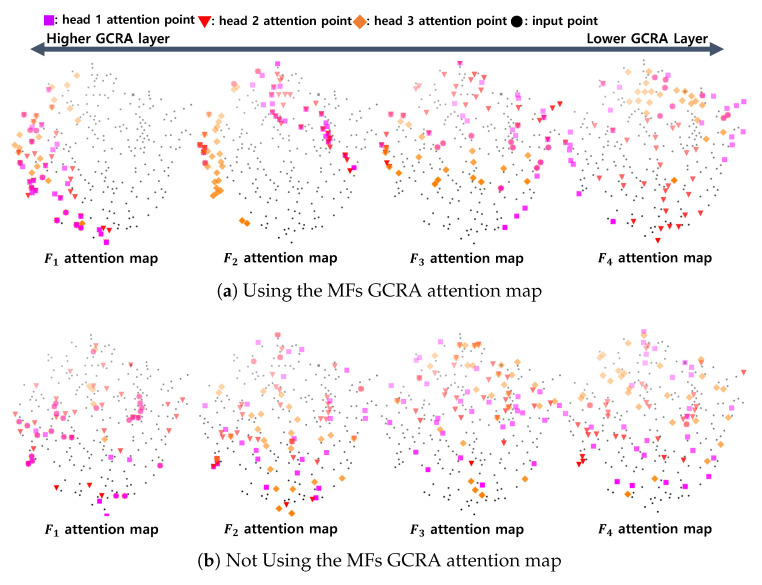
Visualization of attention map generated using MFs as a query in GCR with H=4.

By visualizing the attention maps of all GCRAs, it was confirmed that the GCRAs of GCR with H=4 refined the GFs by adaptively using the MFs extracted from receptive fields of various sizes. Figure 9 shows the results visualized by choosing three attention heads in the GCRA and selecting 30 points, which had the highest attention score in *S*, from each head. The attention map was visualized using Case 3 in Table 4 without MFs in Figure 9b to compare that MFs operated adaptively. As shown in Figure 9a, in the low-layer GCRA, an attention map was formed for a wide range of points in a point set, and in high-layer GCRA, an attention map was formed for a relatively narrow range of points. On the other hand, in Figure 9b, a wide range of attention maps was formed regardless of the high and low levels of the hierarchy. This phenomenon confirmed that PU-MFA uses the adaptive point feature for each layer of the GCRA.

#### 6.3.3. Effect of Noise

**Figure 10:**
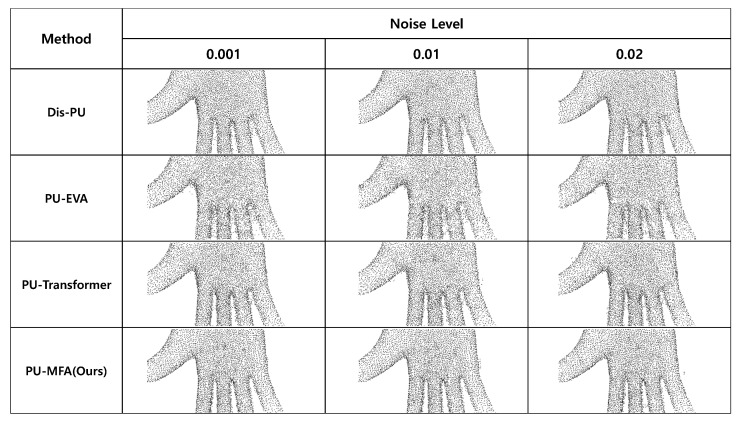
Visualization result of the effect of noise.

Table 5 lists the ×4 up-sampling results of Dis-PU [13], PU-EVA [6], PU-Transformer [5], and the present method using the PU-GAN dataset with various noises added. The noise effect evaluated the result obtained by adding different levels of Gaussian noise N(0,noise level) to a set of input points. As shown in Table 5, the proposed method showed the most robustness to various noise levels. As shown in Figure 10, it can be seen that the boundary between the fingers blurred in the dense set of points generated by the state-of-the-art methods as the noise level was increased. On the other hand, the proposed method showed that the boundary between the fingers was maintained in the dense set of points generated by the present method.

## 7. Conclusions

In this paper, we proposed PU-MFA, a point cloud up-sampling method of U-Net structure that combines multi-scale features and attention mechanism. One of the most significant differences from the prior point cloud up-sampling methods was that PU-MFA used multi-scale features adaptively and effectively through fusion with the cross-attention mechanism. Also, the PU-MFA is the first method to apply the cross-attention mechanism to point cloud up-sampling to the best of the authors’ knowledge. Various experiments were performed on PU-MFA and other state-of-the-art methods using the PU-GAN and the KITTI dataset. As a result, PU-MFA showed better performance of generating high-quality dense point set than other state-of-the-art methods in various experiments. In addition, ablation study showed that multi-scale features are very useful in PU-MFA for generating high-quality point sets by choosing receptive field size adaptively for each layer.

Despite the successful performance at generating high-quality dense point set of PU-MFA, PU-MFA cannot cope with an arbitrary up-sampling ratio. Because PU-MFA is a patch-based up-sampling of ×4, up-sampling is only possible for 4 to the *M* power. A method that can respond to an arbitrary up-sampling ratio is planned in the future to overcome this limitation. 

## Figures and Tables

**Table 1:** Description of symbols.

Symbol	Description
***S***	Sparse point set
$s_{i}$	Element of *S*
$S^{\prime }{}^{\Delta }$	Offset of *S*
$s_{i}^{\prime }{}^{\Delta }$	Element of $S^{\prime }{}^{\Delta }$
*D*	Ground truth point set
$d_{i}$	Element of *D*
$Q^{\prime }$	Coarse point set
$q_{i}^{\prime }$	Element of $Q^{\prime }$
$Q^{\prime }{}^{\Delta }$	Offset of $Q^{\prime }$
$q_{i}^{\prime }{}^{\Delta }$	Element of $Q^{\prime }{}^{\Delta }$
*Q*	Dense point set
$q_{i}$	Element if *Q*
*N*	Input patch size
*r*	Up-sampling ratio
*H*	Depth of layer
$F_{h}$	Set of point wise feature extracted from $h^{th}$ Point Transformer
$f_{i}^{h}$	Point-wise feature extracted from $h^{th}$ Point Transformer
*C*	Channel
$K,K^{\prime }$	Expansion rate
$patch_{i}$	Patch created through KNN based on $s_{i}$
patch_size	Neighbor size of KNN

**Table 2:** Comparing the quantitative evaluation of ×4 and ×16 up-sampling with the state-of-the-art methods.

Method	×4 (2048→ 8192)				×16 (512→ 8192)			
	CD $\left(10^{-3}\right)$	HD $\left(10^{-3}\right)$	P2F $\left(10^{-3}\right)$	#Params (M)	CD $\left(10^{-3}\right)$	HD $\left(10^{-3}\right)$	P2F $\left(10^{-3}\right)$	#Params (M)
Dis-PU	0.2703	5.501	4.346	2.115	1.341	28.47	20.68	2.115
PU-EVA	0.2969	4.839	5.103	2.198	0.8662	14.54	15.54	2.198
PU-Transformer	0.2671	3.112	4.202	2.202	1.034	21.61	17.56	2.202
PU-MFA (Ours)	0.2326	1.094	2.545	2.172	0.5010	5.414	9.111	2.172

**Table 3:** Measure average time complexity after 50 measurements on ×4 up-sampling.

Method	Time per Batch (sec/batch)
Dis-PU	0.02659
PU-EVA	0.02360
PU-Transformer	0.02244
PU-MFA (Ours)	0.02331

**Table 4:** Ablation study results to analyze the effect of the present contribution.

Case	Contribution			Metric		
	MHA	MFE	MFs	CD $\left(10^{-3}\right)$	HD $\left(10^{-3}\right)$	P2F $\left(10^{-3}\right)$
1				0.3349	4.461	4.926
2	*√*			0.2473	1.101	2.829
3	*√*	*√*		0.2500	2.735	2.737
4	*√*	*√*	*√*	0.2362	1.094	2.545

**Table 5:** Quantitative evaluation results of the noise effects using the PU-GAN dataset.

Method	Various Noise Levels Test at ×4 Up-Sampling (CD with $10^{-3}$)					
	0	0.001	0.005	0.01	0.015	0.02
Dis-PU	0.2703	0.2751	0.2975	0.3257	0.3466	0.3706
PU-EVA	0.2969	0.2991	0.3084	0.3167	0.3203	0.3268
PU-Transformer	0.2671	0.2717	0.2905	0.3134	0.3331	0.3585
PU-MFA (Ours)	0.2326	0.2376	0.2547	0.2764	0.2989	0.3195

## Data Availability

Publicly available datasets were analyzed in this study. This data can be found here: https://github.com/liruihui/PU-GAN (accessed date: 12 July 2022).
